# Sensory Reconstruction of the Fossil Lorisid *Mioeuoticus*: Systematic and Evolutionary Implications

**DOI:** 10.3390/ani15030345

**Published:** 2025-01-25

**Authors:** Holly E. Anderson, Adam Lis, Ingrid Lundeen, Mary T. Silcox, Sergi López-Torres

**Affiliations:** 1 University of Warsaw, Faculty of Biology, Żwirki i Wigury 101, 02-096 Warsaw, Polands.lopez-torres@uw.edu.pl (S.L.-T.); 2 Department of Anthropology, Hunter College, City University of New York, 695 Park Ave., New York, NY 10065, USA; 3 Department of Anthropology, University of Toronto Scarborough, 1265 Military Trail, Toronto, ON M1C 1A4, Canada; mary.silcox@utoronto.ca; 4 Division of Paleontology, American Museum of Natural History, 79th Street & Central Park West, New York, NY 10024, USA; 5 Earth Science Department, National Museums of Kenya, Museum Hill, Nairobi 00100, Kenya

**Keywords:** Lorisidae, Kenya, strepsirrhine phylogeny, Miocene, primate, nasal turbinates

## Abstract

The fossil record of lorises and pottos (family Lorisidae) potentially dates back to the late Oligocene of Namibia, but a later moderate diversification of this family occurred during the Miocene of Africa and Asia. In the African Miocene, the family Lorisidae is represented solely by one genus: *Mioeuoticus*. The phyletic position of *Mioeuoticus* has been a source of debate, as it has been suggested to belong to either the stem of the family Lorisidae or to be further nested within lorisids, as a sister to the African potto clade (subfamily Perodicticinae). Reconstructing the internal sensory anatomy of *Mioeuoticus shipmani* (KNM-RU 2052) could shed some light on this debate and possibly clarify how modern lorisoid olfactory and visual sensitivity and locomotor abilities evolved. Here, we collected data from the nasal turbinals, bony labyrinths, and orbits of *Mioeuoticus shipmani* from the early Miocene of Rusinga Island, Kenya. These results are consistent with *Mioeuoticus*, having developed typical modern lorisid behaviour (i.e., slow locomotion, nocturnal activity pattern) and olfactory abilities consistent with modern representatives. However, the arrangement of the nasal turbinals shows an intermediate state between lemuroids and lorisoids that is most consistent with a basal position of *Mioeuoticus* within the family Lorisidae or even the superfamily Lorisoidea.

## Simple Summary

We reconstructed the internal sensory system anatomy of the lorisid, *Mioeuoticus shipmani* (KNM-RU 2052), from the early Miocene of Rusinga Island, Kenya. Results suggest that *Mioeuoticus* developed typical modern lorisid behaviour (i.e., slow locomotion, nocturnal activity pattern) and olfactory abilities consistent with modern representatives.

## 1. Introduction

Lorises and pottos (family Lorisidae) are nocturnal strepsirrhine primates that live in equatorial Africa, and southern and southeastern Asia. Compared to other primate families, lorisids are not a particularly speciose clade [1] but became highly specialised to a nocturnal, arboreal lifestyle and insectivorous dietary behaviours, often showing adaptations for gum extraction [2,3,4]. There has been extensive research into the ecology, behaviours, and evolutionary relationships of the modern lorisoids. The paucity of these animals in the fossil record means that our understanding of lorisid phylogeny, systematics, reconstructed behaviour, and functional anatomy in these fossil relatives is comparatively scarce and major aspects remain unresolved [1]. However, this is not to say there have not been significant discoveries from the available fossil specimens, which have significantly advanced our knowledge of lorisoid evolutionary histories, such as the potential origins of lorisoids [5] and lorisids [6], certain aspects of their locomotor ecology [7], and identification of a greater diversity of dietary behaviours in extinct forms [8,9,10,11,12]. The fossil discoveries that have been made clarify the interrelationships of extant lorisid genera and reveal seemingly good convergence between fossils and molecular divergence date estimates in certain parts of the phylogenetic tree [1].

Despite these advances, many questions remain as to the reconstructed behaviour in fossil lorisid forms. Whereas most fossil lorisid material comprises dentognathic or, occasionally, postcranial material [13,14], an almost complete cranium is known from the early Miocene of Rusinga Island, Kenya, belonging to *Mioeuoticus shipmani* [15]. The phylogenetic position of *M. shipmani* is currently uncertain, with Walker et al. [7] placing it as a sister group to all African lorisids (i.e., subfamily Perodicticinae), while Harrison [14] argued that the genus *Mioeuoticus* belongs to a more basal lineage at the stem of lorisids. In either case, *Mioeuoticus* represents an early member of the family Lorisidae and understanding its behaviour provides a deep evolutionary perspective in the context of the clade. The reconstructed behaviour of *M. shipmani* remains largely unexplored, but it is not completely unknown. Walker et al. [7] found that the semicircular canals (SCCs) in *M. shipmani* had a similar relative size to those seen in modern slow lorises (genus *Nycticebus*). The SCCs provide information on the body’s position in space, contributing in particular to the stabilisation of gaze in locomotion [16]. Among modern primates, slower-moving species tend to have smaller SCCs for a given body mass compared to faster-moving taxa [16]. The similarities between *Mioeuoticus* and *Nycticebus* found by Walker et al. [7] suggest, therefore, that *Mioeuoticus* was most likely a slow-moving arboreal quadruped, in a similar fashion to modern-day lorisids. However, besides the reconstructed locomotor behaviour, nothing is known about other aspects of its behaviour and, in particular, its sensory capabilities.

The main aim of this project is to reconstruct the sensory capacities of the early Miocene lorisid *Mioeuoticus*. The relative timing of the evolution of these characteristics may have important implications for the phyletic position of the early Miocene lorisid *Mioeuoticus*.

### 1.1. Vision

The optic foramen quotient (OFQ) value calculated for any given primate acts as a proxy for the degree of retinal summation, which is a quantification of the number of photoreceptor cells that converge on bipolar cells in the eye to transmit signals to ganglion cells for transfer to the brain for interpretation and response [17]. This interpretation and response deduces how to interact with and adapt to various life-preserving factors, including predators, food, and long-distance conspecific communication [18,19,20]. Thus, the OFQ gives a solid proxy for the degree of visual acuity in a primate; a low OFQ means high central retinal summation and a high OFQ means low central retinal summation [21].

The dimensions of the intra-orbital surface can be directly related to the size of the optic nerve in primates [22]. Direct comparisons with optic nerve measurements in extant primates with known visual capacities can provide a basis to hypothesise the visual acuity of fossil primates [21]. The left intra-orbital surface of the *Mioeuoticus* cranium volume rendering has been significantly taphonomically distorted; however, the right intra-orbital surface appears to be well preserved with little to no apparent damage.

### 1.2. Olfaction

To ensure survival, mammals rely strongly on their sense of smell to convey critical information about food seeking, mate selection, hazard detection, and behavioural immune responses [23,24,25,26,27,28,29]. The olfactory system is structured as long, scrolled, spongy networks of bone within the nasal cavity, known as turbinals, turbinates, or conchae. The turbinals are lined with a specialised tissue called olfactory epithelium, which contains receptor cells that bind with molecules that detect odorants that the brain interprets as smell [30,31]. Thousands of chemically diverse odour molecules and pheromones are detected and recognised by the olfactory receptor neurons in the olfactory epithelium, which are converted into electrical signals that are sent to the brain for interpretation and behavioural response [32,33]. Many primates also have a vomeronasal organ, with the purpose of detecting pheromones critical in perceiving and processing stimuli related to social and reproductive behaviours [34,35].

The proportions, number, and complexity of these structures give strong evidence of the olfactory landscape, behavioural ecology, and phylogenetic affinities of fossil primates [36,37,38,39,40]. These data may detect a system specialised to a specific range of odorants, which can strongly indicate the primate trends in the evolution of nasal morphometry and airflow [38,41,42]. Turbinals are delicate and rarely preserved in fossil specimens, limiting opportunities to make direct observations of the olfactory periphery in extinct primates. Fortunately, the turbinals of the *Mioeuoticus* specimen in this study are preserved with minimal damage or distortion.

### 1.3. Audition and Proprioception

Mammals depend on audition for vital interactions with their environments, including food detection and attainment [43,44], hazard detection and evasion [45,46], stabilising balance [16], and participating in communal exchanges governing territoriality or mating [47,48]. The auditory system comprises two fluid-filled receptor organs that form the membranous labyrinth, which is suspended within the petrous temporal bone inside a network of bony voids known as the bony labyrinth [49]. The bony labyrinth consists of semicircular canals (SCCs), the utricle and the sacculus, and the cochlea.

As sound waves enter the inner ear, the fluid in the utricle, sacculus, and cochlear receptor organs vibrate. The hairs in the cochlea have different lengths and vibrate at numerous sound frequencies. Stimulating these microscopic hairs generates electrical impulses and sends them to the brain for interpretation and behavioural response [7,50]. The semicircular canals regulate balance and sense head position to maintain stability despite motion and activity. The orientation, relative position, and size of the semicircular canals give strong evidence of the locomotor behaviour and shape concerning phylogenetic affinities of fossil primates [51,52,53,54,55,56,57]. Measurements of semicircular canals in extant mammals with known locomotor behaviours can provide a basis for direct comparisons with which to test hypotheses about locomotion in fossil primates [58,59]. The bony labyrinths of the *Mioeuoticus* specimen in this study are difficult to quantify due to poor CT resolution, but some diagnostic structures appear to be preserved with minimal damage or distortion.

### 1.4. Phylogenetic Implications

These data may provide critical insights as to whether *Mioeuoticus* are stem or crown members of Lorisidae, or possibly advanced stem, or very basal crown, lorisoids. To better understand the phylogenetic and ecological significance of these observations for *Mioeuoticus*, we provide a comparative framework with analyses of extant and extinct specimens in the associated literature for evaluating sensory character evolution to assess the relationships between fossil and living lorisids. Understanding how fossil lorisids relate to their living relatives is critical for explaining major trends in primate evolution. A better understanding of the relationships among extinct and extant lorisids may speak to the question of the biogeographic origin of this primate family and a broader understanding of the characteristic low diversity of modern lorisids.

### 1.5. Institutional Abbreviations

BAA—Department of Evolutionary Anthropology, Duke University, Durham, NC, USA; MCZ—Museum of Comparative Zoology, Harvard University, Cambridge, MA, USA; AMNH—American Natural History Museum, NY, USA; YPM—Yale Peabody Museum at Yale University, New Haven, CT, USA; KNM-RU—National Museums of Kenya, Rusinga Island collection, Nairobi, Kenya.

## 2. Methods

**Figure 1:**
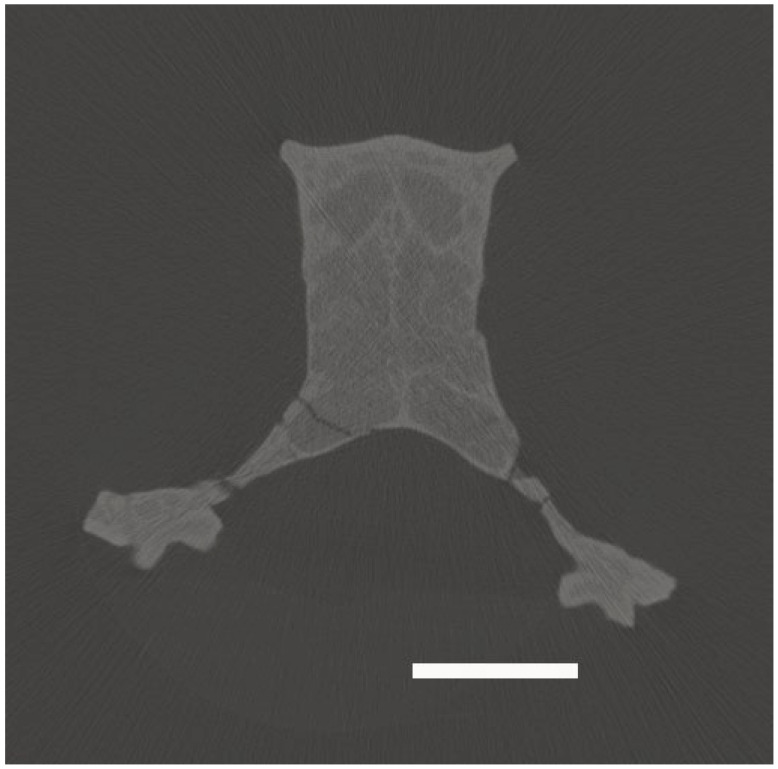
Tiff file image (slide 178) showing the olfactory cavity of *Mioeuoticus shipmani* (KNM-RU 2052; holotype), highlighting the poor resolution of the sole cranial scan of this species. Scale bar is 5 mm.

The cranium of *Mioeuoticus shipmani* (KNM-RU 2052; housed in the Palaeontological Collections of the National Museums of Kenya, Nairobi) had been previously scanned on-site using a portable micro-CT scanner by a team from Kyoto University. The resulting scan has 575 total slices with voxel dimensions of x = 0.055, y = 0.07, and z = 0.07, providing a 16-bit TIFF image stack data set for the cranium, and the slices were rendered as 3D volumes in Avizo 3D Pro 2023 (Visualization Sciences Group, Berlin, Germany). This three-dimensional rendering was used to reconstruct the internal anatomy of the sensory systems as digital endocasts. These structures of *Mioeuoticus* (as evident in Figure 1) were manually segmented in each slice using the brush tool.

### 2.1. Vision

The visual acuity of *Mioeuoticus shipmani* is analysed following the OFQ method and analysis of the relationship between orbit diameter and cranial length, and activity period, as described by Kirk and Kay [21]. The length and width of the orbital and optic foramen cavities in the three-dimensional volume rendering of the *Mioeuoticus* cranium are taken using the ruler tool on Avizo 3D (See Supplementary Table S1); thus, the area and diameter of these two regions can be calculated. The orbit diameter is 16.52 mm and the P-I length is 60.33 mm. When compared to the bivariate plot of ln mean orbit diameter and ln mean cranial length for 60 extant primate species in Figure 5B of Kay and Kirk [17] *Mioeuoticus shipmani* lies firmly in the group with nocturnal strepsirrhines.

The formula for the optic foramen area is then calculated as follows: A = π a b where A is the area of the orbital cavity, a is the major radius length, and b is the minor radius length of the optic foramen and orbital cavity. We then measure from the craniometric point, which is the most anterior point in the midline on the alveolar process of the maxilla, to the tip of the external occipital protuberance—known as the prosthion–inion (P-I) length. This calculation is used as a substitute and estimation for the total body size of this specimen.

These data are then added as variables into the equation by Kirk and Kay [21] to approximate optic foramen size relative to eye size, as follows:

(Optic Foramen Area/Orbit Area) × 100 = Optic Foramen Index (OFI)

Kirk and Kay [21] state that this equation increasingly overestimates eye size as the body size of a specimen increases, and thus to remove this bias, the OFI result for body size must be adjusted using the following equation:

OFQ = (Observed OFI-Expected OFI)/Expected OFI] × 100.

We then measured the prosthion–inion (P-I) length, which runs from the craniometric point, which is the most anterior point in the midline on the alveolar process of the maxilla, to the tip of the external occipital protuberance (see Supplementary Table S1). P-I length is needed to calculate the expected OFI.

The expected OFI is calculated with two empirical regression formulas to express the OFI as a percentage of that which is expected for diurnal anthropoids (LnOFI = 3.39983 − 0.56624 × ln [P-I length]) and nocturnal strepsirrhines (LnOFI = 2.46913 − 0.58138 × ln [P-I length]) of similar P-I length. Therefore, we input the P-I length of the *Mioeuoticus* holotype into both of these equations and raise the result of these calculations to the power of e, where e ≈ 2.718, to determine the diurnal anthropoid regression (daOFQ) and nocturnal strepsirrhine regression (nsOFQ) for the specimen, as follows:

Diurnal Anthropoid Expected OFI: LnOFI = 3.39983 − 0.56624 × Ln (P-I length)

daOFQ = (Observed OFI-Expected OFI)/Expected OFI] × 100

Nocturnal strepsirrhine Expected OFI: LnOFI = 2.46913 − 0.58138 × Ln (P-I length)

nsOFQ = (Observed OFI-Expected OFI)/Expected OFI] × 100

These data were then directly compared to the daOFQ and nsOFQ of extant specimens determined in Table 5 of Kirk and Kay [21] to assess visual acuity in *Mioeuoticus* (see Supplementary Table S2).

### 2.2. Olfaction

We provide a detailed description of the reconstructed *Mioeuoticus* olfactory turbinals, in which all nomenclature and definitions of turbinals follow Smith and Rossie [60]. We follow the convention of numbering ethmoturbinals using Roman numerals from anterior to posterior view. All descriptions of turbinal morphology follow an anterior-to-posterior sequence based on successive coronal CT slices. Terms used to describe turbinal shape in coronal cross-section follow Lundeen [41]. When possible, turbinals were segmented from the lateral wall of the nasal fossa at the point where the turbinal’s basal lamina make contact with the lateral wall. We also quantify the surface areas of both the olfactory turbinals and the maxilloturbinals of the *Mioeuoticus* specimen using the surface area tool in Avizo. The overall turbinal and maxilloturbinal data are both correlated with turbinal surface areas of a range of extant strepsirrhines and haplorhines calculated by Lundeen and Kirk [38] to hypothesise the olfactory abilities of *Mioeuoticus* compared to other similarly sized primates.

### 2.3. Audition

We provide detailed descriptions of the overall morphology of the bony labyrinth, focused mainly on the orientation, relative position, and size of the semicircular canals. The anterior, posterior, and lateral semicircular canals are described, respectively, as they appear in anterior-to-posterior view based on successive coronal CT slices. The angle between the three canal arcs is measured at the greatest width of the anterior and posterior semicircular canals on the transverse plane, following Spoor and Zonneveld [53].

The medial end of the anterior (superior) semicircular canal joins with the upper part of the posterior canal to form a bony limb, known as the common crus [61,62], whose length we additionally measure (CCL). CCL is measured from the opening of the vestibular aqueduct in the vestibular wall to the bifurcation point of the common crus—known as the crus commune apex—following inner ear morphological landmark locations defined in LeBrun [61]. The length of the common crus in the reconstructed *Mioeuoticus* inner ear is compared to the mean CCL in extant slow- and fast-moving primates (Pier et al. [62]: Table 4).

## 3. Results

The cranial measurements of the holotype of *Mioeuoticus shipmani* (KNM-RU 2052) are as follows: prosthion–inion length of 61.8 mm, cranial height of 26.3 mm, neurocranial width of 33.9 mm, and maxillary width of 30.5 mm. Cranial height is measured from the basion (midpoint of the anterior margin of the foramen magnum) to the highest point on the skull. Neurocranial width is measured across the most lateral aspect of each parietal (euryon to euryon). Maxillary width is measured across the most lateral aspect of each temporal bone.

### 3.1. Vision

Following the OFI and OFQ equations of Walker et al. [7], the daOFQ and nsOFQ were calculated for the holotype of *Mioeuoticus shipmani*.

Prosthion–Inion (P-I) length = 60.33

LnP-ILength = 4.099829492

daOFQ:

= 3.39983 − (0.56624 × LnP-ILength) = 1.078342548

= Exp(1.078342548) = 2.939802929

= ((1.273915072 − 2.939802929)/2.939802929) × 100

= −56.666651

NsOFQ:

= 3.39983 − (0.56624 × LnP-ILength) = 0.085899116

= Exp(0.085899116) = 1.08969639

= ((1.273915072 − 1.08969639)/1.08969639) × 100

= 16.90550541

### 3.2. Olfaction

Detailed morphological analyses and anatomical measurements for the individual turbinals in the *Mioeuoticus* olfactory reconstruction are presented below. These data are likely an underestimation as fragmentation of the turbinals reduces their overall surface area to less than that of the original total turbinal structure and the turbinal surface area present in the living *Mioeuoticus* nasal cavities, though still provide a novel physiological understanding of this previously unstudied olfactory system. The internal turbinal measurements are available in Table 1.

**Figure 2:**
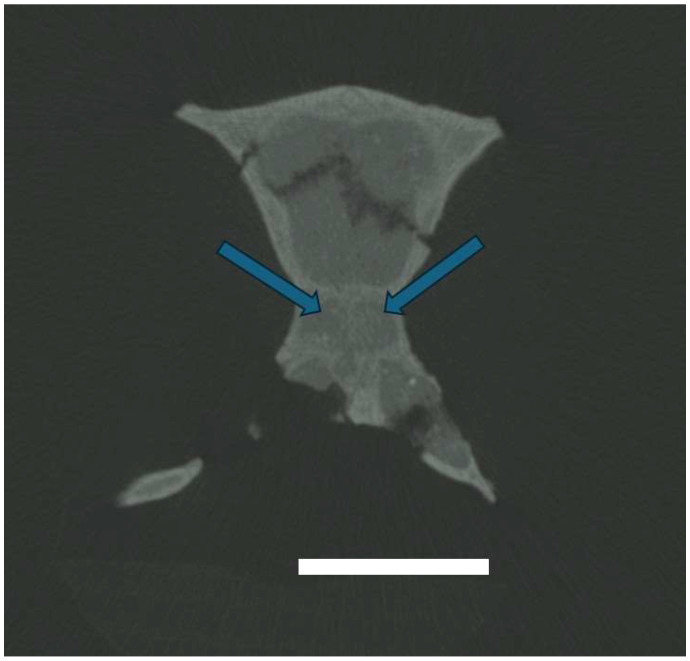
CT scans (slide 208) show the olfactory recess of *Mioeuoticus shipmani* (KNM-RU 2052; holotype), with blue arrows highlighting the change in resolution that suggests the presence of a fourth pair of ethmoturbinals. Scale bar is 5 mm.

**Figure 3:**
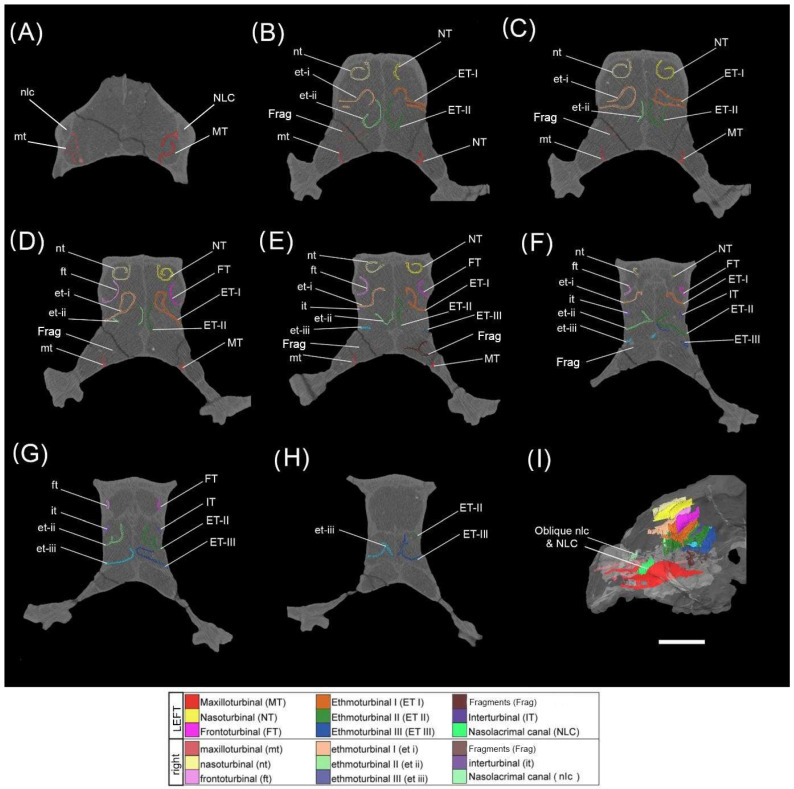
CT scans through the nasal fossa of the holotype of *Mioeuoticus shipmani* (KNM-RU 2052). (**A**–**H**) Slices along the coronal plane showing preservation of left and right nasal fossae: slice number 97 (**A**), 137 (**B**), 139 (**C**), 146 (**D**), 156 (**E**), 173 (**F**), 179 (**G**), and 190 (**H**) of 449 total slices. (**I**) Digitally bisected volume renderings of the cranium showing posterior nasal fossa anatomy in *Mioeuoticus*. Frag = unidentified floating turbinal fragment. Scale bar is 10 mm.

The cross-sectional morphology of each of the turbinals is described below from anterior to posterior on a coronal plane (Figure 2 and Figure 3), including anatomical details of the olfactory cavity, the relative position of each turbinal in the nasal fossa, and the nature of the turbinals’ contact with the fossa wall or other turbinals. It is recognised that debate still lies in whether to group the two closely associated anterior-most turbinal structures as a single ethmoturbinal (ethmoturbinal I) or divide them into two ethmoturbinals (ethmoturbinal I and II). Here, we take a conservative approach and divide these structures into two ethmoturbinals. In doing so, we form a comprehensive description of each structural subdivision that may better contribute to future evaluations of these characteristics within the appropriate comparative context. Turbinal names are abbreviated as follows (left nasal turbinals—uppercase abbreviations; right nasal turbinals—lowercase abbreviations); NT/nt—nasoturbinal, FT/ft—frontoturbinal, ET/et-I to -IV—ethmoturbinals 1 to 4, IT/it—interturbinal, MT/mt—maxilloturbinal.

The *Mioeuoticus* specimen has all the turbinals we may expect to find here and there is a symmetry between the left and right fossa, though these turbinals are broken and fragmented. In total, there is one pair of nasoturbinals, frontoturbinals, interturbinals, and maxilloturbinals, and three pairs of ethmoturbinals. The left and right turbinals are separated in the anterior portion of the olfactory cavity by the nasal septum. In the posterior of the olfactory cavity, a transverse septum separates a superior olfactory recess from an inferior nasopharyngeal meatus enclosing all of the turbinals.

There may also be a fourth pair of ethmoturbinals in the olfactory recess of this specimen, though this cannot be fully confirmed nor reconstructed due to the poor resolution of the scan (Figure 2). No defining structure can be seen, and so these turbinals are not described any further below.

#### 3.2.1. Turbinals of the Left Nasal Cavity

The left nasal turbinals are well preserved overall, with all major turbinals mostly intact. The left nasal fossa contains one NT, one FT, three ETs, one IT, and one MT.

There is a minor mediolateral distortion of the NT, and ET-I, -II, and -III.

**Figure 4:**
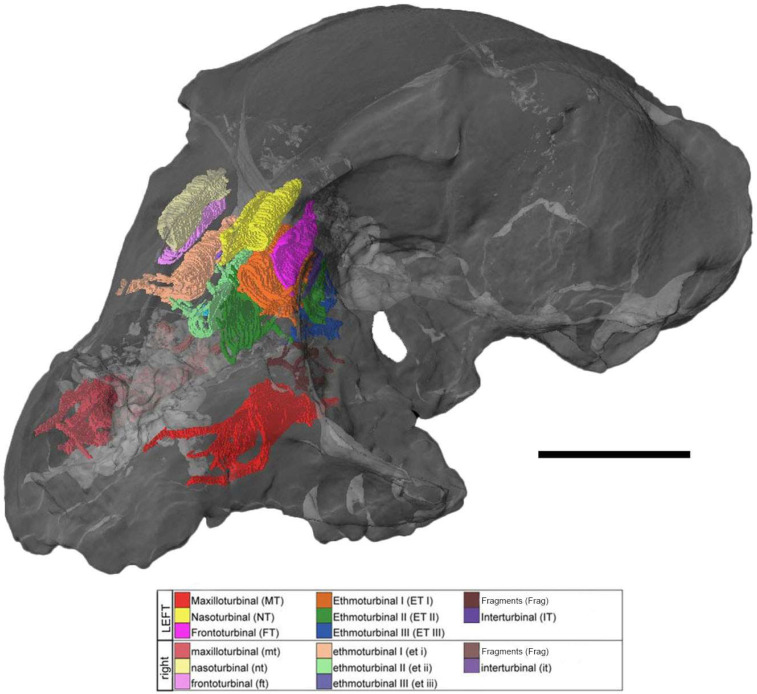
Three-dimensional volume rendering of the cranium of *Mioeuoticus shipmani* (KNM-RU 2052; holotype) in oblique left lateral view. Nasal turbinals have been reconstructed. Scale bar is 10 mm.

**Figure 5:**
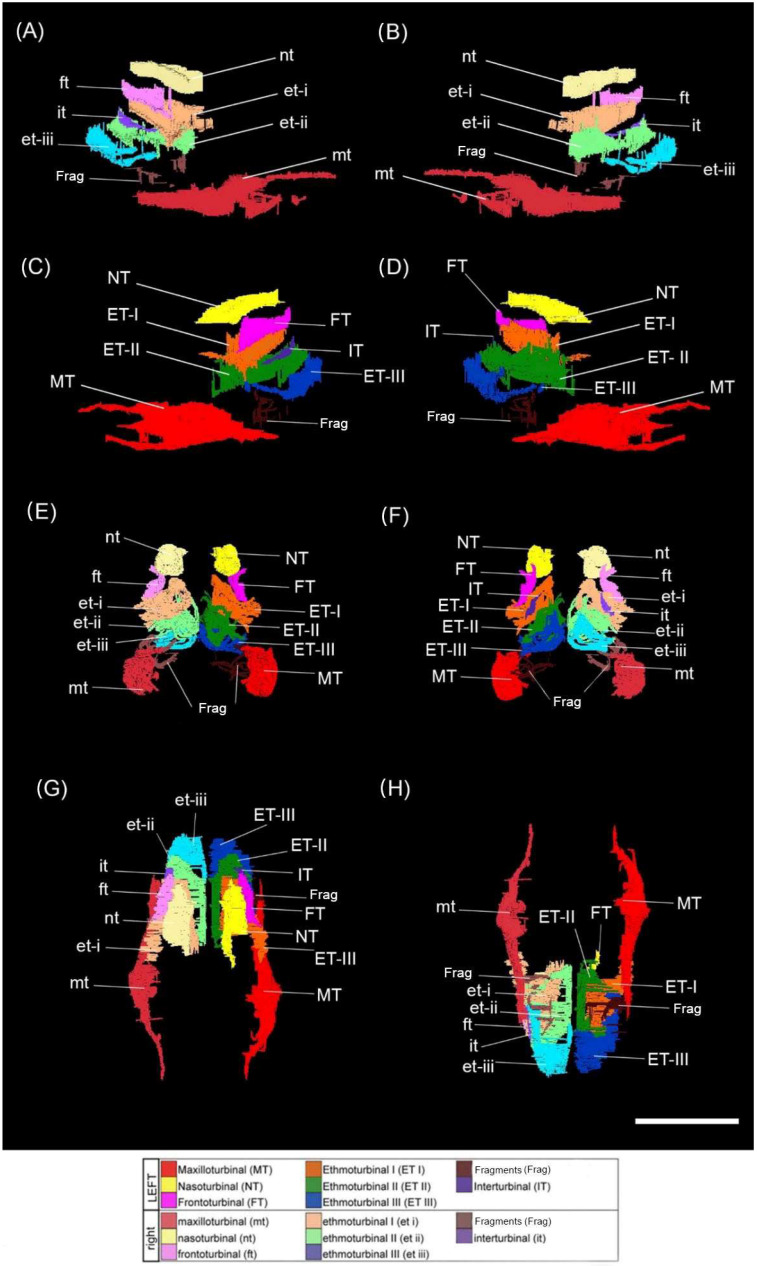
Turbinals of the holotype of *Mioeuoticus shipmani* (KNM-RU 2052) in (**A**) right lateral view; (**B**) medial view of right nasal fossa turbinals; (**C**) left lateral view; (**D**) medial view of left nasal fossa turbinals; and (**E**) anterior, posterior (**F**), superior (**G**), and inferior (**H**) views of all turbinals. Right (uppercase abbreviations) and left (lowercase abbreviations) sides in surface view can be compared with cross-section views in Figure 1. Frag = unidentified floating turbinal fragment. Scale bar is 10 mm.

*Maxilloturbinal* (*MT*)—This turbinal is the most anterior of the assemblage, situated only 1.3 mm posterior to the piriform aperture, with a slight posterior sloping gradient on the sagittal plane. This turbinal’s anterior and inferior margins are preserved along the full length of the structure. This is a double-scrolled turbinal, with both the medially and laterally directed scrolls well preserved (as seen in slice 97 of the specimen CT scan; Figure 3A, Figure 4, and Figure 5C–H). It is difficult to assess whether the anterolateral wall of the turbinal ceases at the scrolled portion of the structure or has been destroyed and would extend to the full extent of the basal lamina.

*Nasoturbinal (NT)*—This turbinal is a single-scrolled structure that appears almost perfectly preserved (as seen in slices 137, 139, 146, and 156 of the specimen CT scan; Figure 3B–E, Figure 4, and Figure 5C–G). There is no apparent deformation, fragmentation, or destruction. The turbinal is orientated anteromedially in the olfactory cavity with a significant anteriorly sloping gradient, 15.4 mm posterior to the piriform aperture. The anterior-most margin of the turbinal is anterolateral to the scrolled midsection, which has a ‘teardrop’ shape in the cross-section. The turbinal has a single direct point of contact with the superior fossa wall running anteroposteriorly.

*Ethmoturbinal I (ET-I)*—This turbinal is positioned inferiorly to the NT and FT 13.4 mm to the piriform aperture, with a steep anterior gradient on the sagittal plane. It is on the same sagittal plane as the NT and inferolateral to the FT. Anteriorly, the ET is broken. Further towards the central structure, the ET forms a single bulla for the main portion of the turbinal, which detaches laterally and arcs into a scroll that reduces to a branching structure. This enclosed bulla decreases in volume posteriorly. Initially, the enclosed bulla has two points of contact with the lateral fossa wall. As the bulla detaches to a scroll structure, these connections merge into a singular point of contact (as seen in slices 139 and 146 of the specimen CT scan; Figure 3C,D, Figure 4 and Figure 5A–F).

*Ethmoturbinal II (ET-II)*—This turbinal is inferomedial to ET-I, situated 13.1 mm posterior to the piriform aperture, and orientated anteromedially in the olfactory cavity with a steep anterior gradient on the sagittal plane. Anteriorly, ET-II is a singular bulla that terminates at two points of contact with the inferior medial and lateral border of ET-I (as seen in slice 137 of the specimen CT scan; Figure 3B). The medial point of contact with ET-I disconnects posteriorly, forming an arcuate structure that extends horizontally across the nasal fossa and then curves upward to run parallel with the septal wall (as seen in slice 139 of the specimen CT scan; Figure 3C). It is unclear whether the terminal margin of the bulla is incomplete or the resolution of the scan is too poor to discern it. The connection with the superior fossa wall is lost towards the main portion of this turbinal (as seen in slices 146, 156, and 173 of the specimen CT scan; Figure 3D–F, Figure 4 and Figure 5A–F). Anteriorly, the turbinal is a branching structure that contact the superior and lateral fossa wall (as seen in slice 179 of the specimen CT scan; Figure 3G).

*Frontoturbinal (FT)*—This turbinal is located inferolateral to the ET-II, orientated anteromedially in the olfactory cavity with a steep anterior gradient on the sagittal plane. Situated 16.4 mm posterior to the piriform aperture, it has an arcuate morphology; a regular arc that increases in circumference from anterior to posterior (as seen in slices 146, 156, and 173 of the specimen CT scan; Figure 3D–F and Figure 5E–G). It cannot be determined whether this is due to damage and fragmentation of a scroll or if this is the original structure. The turbinal has a single direct point of contact with the lateral fossa wall running anteroposteriorly.

*Ethmoturbinal III (ET-III)*—Anteriorly, this turbinal is inferior to ET-II, located on the same sagittal plane in the anterior, at 13.1 mm posterior to the piriform aperture. Posteriorly, the turbinal is lateral to ET-II as it is orientated anteromedially in the olfactory cavity with a steep anterior gradient on the sagittal plane. It is difficult to discern whether this turbinal is bullar (via a superior and inferior contact with the lateral fossa wall) or arched (with a single point of contact with the fossa wall), as part of ET-III is not visible on the CT rendering. Posteriorly, this arch curves more steeply to form a single medial scroll with branches that contact the septal wall (as seen in slice 179 of the specimen CT scan; Figure 3G). More superiorly, a superior branching point of contact is also formed (as seen in slice 190 of the specimen CT scan; Figure 3H). The scroll gradually becomes less pronounced, and the medial branch extends until the turbinal becomes a single superior branching point of contact that descends along the centre of the nasal fossa and splits to form two equal curved branches in contact with the lateral and medial septal wall.

*Interturbinal (IT)*—This turbinal is the most posterior of the assemblage, located inferolateral to the FT, with a gradual anterior gradient on the sagittal plane. It is located 19.6 mm posterior to the piriform aperture, with an arcuate branching structure that projects from the lateral fossa wall between ethmoturbinal I and II. This turbinal arches, mirroring the curvature of the frontoturbinal (as seen in slices 156, 173, and 179 of the specimen CT scan; Figure 3E–G).

#### 3.2.2. Turbinals of the Right Nasal Cavity

The right nasal fossa contains one nt, one ft, three et, one it, and one mt. The right nasal turbinals are well preserved overall, with all major turbinals mostly intact. There is a minor mediolateral distortion of et -I, -II, and -III.

*Maxilloturbinal (mt)*—The structure of this turbinal closely mirrors that of the paired MT in structure and position, located 0.7 mm posterior to the piriform aperture. The basal margins are once again mostly preserved along the turbinals full length, though the anterior margin is comminuted - as are the medially and laterally directed scrolls (as seen in slice 97 of the specimen CT scan; Figure 3A). The laterally directed scroll has begun to degrade into floating fragments.

*Nasoturbinal (nt)*—This turbinal closely matches the structure and position of the paired NT and is also well preserved, other than anterior deformation of the laterally directed scroll along the coronal plane (as seen in slices 137, 139, 146, and 156 of the specimen CT scan; Figure 3B–E). The turbinal is located 15.6 mm posterior to the piriform aperture.

*Ethmoturbinal I (et-I)*—The ET-I and et-I are similar, though the bulla of et-I does not detach to form a scroll which then reduces to a branching structure; instead, it detaches immediately from a bulla to branching morphology from the middle of the structure to the posterior (as seen in slices 139 and 146 of the specimen CT scan; Figure 3C,D and Figure 5A–F). The turbinal is positioned 12.2 mm from the piriform aperture. Furthermore, the bulla is much narrower in the cross-section and more elliptical than in ET-I.

*Ethmoturbinal II (et-II)*—This turbinal shares the same general arrangement as ET-II and is positioned similarly 14.1 mm from the piriform aperture. The full extent of the turbinals is not observable, either due to damage or poor CT resolution. The ET-II and et-II are almost identical anteriorly, as a branching structure in contact with the superior and lateral fossa wall (as seen in slices 146, 156, and 173 of the specimen CT scan; Figure 3B–F and Figure 5A–F). However, the two points of contact of the et-II bulla with the inferior lateral border of ET-I are more lateral and superior, respectively. The bulla is also much more convex posteriorly (as seen in slice 146 of the specimen CT scan; Figure 3D and Figure 5E–G).

*Frontoturbinal (ft)*—Similarly to FT, the ft has an arcuate morphology—a regular arc that increases in circumference anteroposteriorly (as seen in slices 137, 139, 146, 156, and 173 of the specimen CT scan; Figure 3D–F). The arch is more convex, however, which may be a natural disparity or possibly due to mediodorsal compression and deformation. This turbinal is slightly more posterior than its counterpart, 18.4 mm from the piriform aperture.

*Ethmoturbinal III (et-III)*—This turbinal is almost identical to ET-III, other than that a greater portion of the morphology is not evident in the scan, either due to damage or poor CT resolution (as seen in slice 179 of the specimen CT scan; Figure 3G). It is difficult to assess whether the turbinal naturally ceases at the scrolled portion of the structure or is fragmented and would extend to a complete bulla (as seen in slice 190 of the specimen CT scan; Figure 3H and Figure 5C,D,H). It is similarly located to its opposing paired turbinal, 12 mm posterior to the piriform aperture.

*Interturbinal (it)*—This turbinal is almost completely identical to its right pair, though the anterior segment which extends anteromedially is slightly shorter (as seen in slices 156, 173, and 179 of the specimen CT scan; Figure 3E–G and Figure 5A–H). The positioning is almost identical too, 18.6 mm posterior to the piriform aperture. It is difficult to distinguish whether this is a natural variation or due to some minor destruction of the turbinal.

Numerous floating fragments are evident in both anterior nasal fossae. Due to severe taphonomic alterations, there is little evidence that can be derived from these fragments, and it cannot be determined which of the turbinals they originate from.

The total surface area of the *Mioeuoticus* specimen’s reconstructed olfactory assemblage is 1109.92 mm^2^, though it is likely that this is an underestimation as breakage and fragmentation are evident in every turbinal structure. A further estimate of total surface area which may be more representative is deduced by considering only the most complete turbinal from each pair and multiplying these data by two, the result of which is 1184 mm^2^. The surface areas of the individual olfactory components are summarised in Table 2. The maxilloturbinals, determined from the average of each paired olfactory component, comprise the most substantial portion (26%) of the total surface area. This is followed by ethmoturbinals I (21%) and nasoturbinals (15%), ethmoturbinals II (18%), ethmoturbinals III (11%), frontoturbinals (6%), the remaining floating fragments (4%), and, finally, the interturbinals (2%).

#### 3.2.3. Vomeronasal Groove

**Figure 6:**
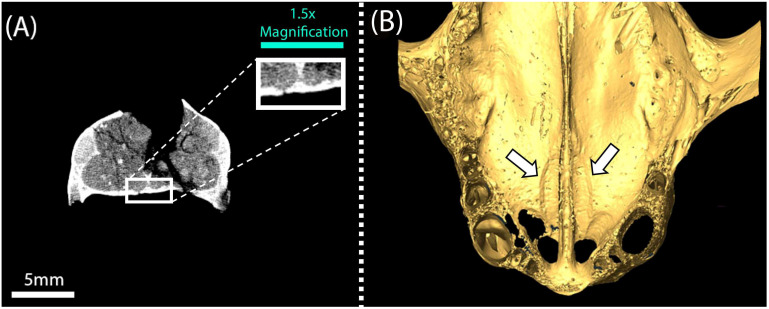
(**A**) A two-dimensional CT scan (slide 64) shows a coronal section of the cranium of *Mioeuoticus shipmani* (KNM-RU 2052) at the level of P_2_ with a vomeronasal groove on the base of the olfactory cavity. The boxed area of the image is magnified 1.5× in the top-right corner for comparison and clarity. (**B**) Dorsal view of the base of the olfactory cavity of *Perodicticus ibeanus* (AMNH-52682) with a pair of vomeronasal grooves. Vomeronasal grooves are indicated with white arrows. White scale bar is 5 mm. Blue scale bar at 1.5× magnification is 3.3 mm.

An additional significant observation made when analysing the inferior base of the rostral cranium of the *Mioeuoticus* specimen is the vomeronasal groove (Figure 6). The vomeronasal groove is a trough-like structure (e.g., Figure 6A) that houses the cartilaginous tube of the vomeronasal organ, which sits at the base of the nasal cavity anteroventrally to the septum (e.g., Figure 6B) and is covered in odorant receptors [63,64,65,66].

Modern strepsirrhines possess a vomeronasal organ [34,35,41,67,68,69], though due to the endangered nature of many strepsirrhine taxa, anatomical analysis is restricted to only a small number of taxa [70,71,72,73,74,75]. Even rarer are the studies to reconstruct the vomeronasal organ of extinct strepsirrhines using modern comparatives [66], and anatomical analysis of fossil primate vomeronasal grooves [76]. This study constitutes the oldest evidence of the vomeronasal groove in a fossil crown strepsirrhine and certainly is the first record of this characteristic in fossil lorisoids.

### 3.3. Audition

**Figure 7:**
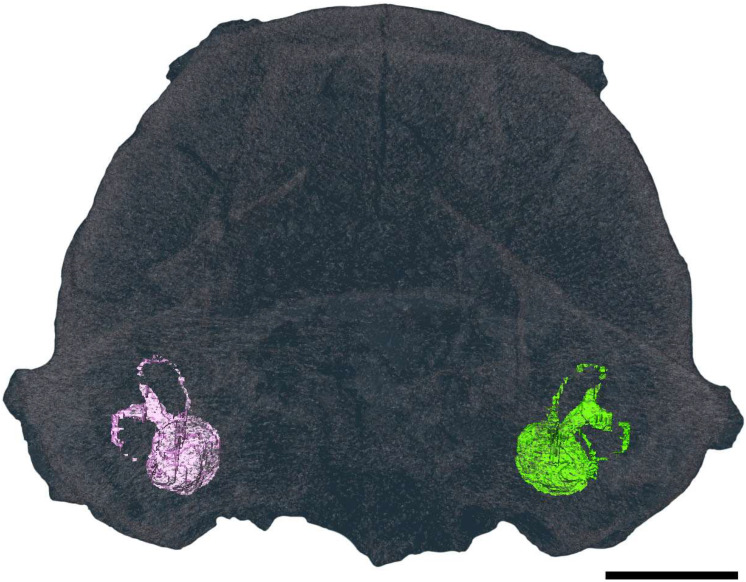
Reconstructed left (pink) and right (green) bony labyrinths of the holotype of *Mioeuoticus shipmani* (KNM-RU 2052) in posterior view of the cranium. Scale bar is 5 mm.

The general morphology of the cochlea is evident, but the coiling structure cannot be discerned in the cast. The length of the cochlear canals from the apex of the spiral to the terminal base (near the middle ear and the oval window) can be measured at 3.94 mm (left) and 4.02 mm (right). The three oval semicircular canals (SCCs) are apparent in both bony labyrinths: the anterior semicircular canal (ASC), the posterior semicircular canal (PSC), and the lateral semicircular canal (LSC) (Figure 7).

**Figure 8:**
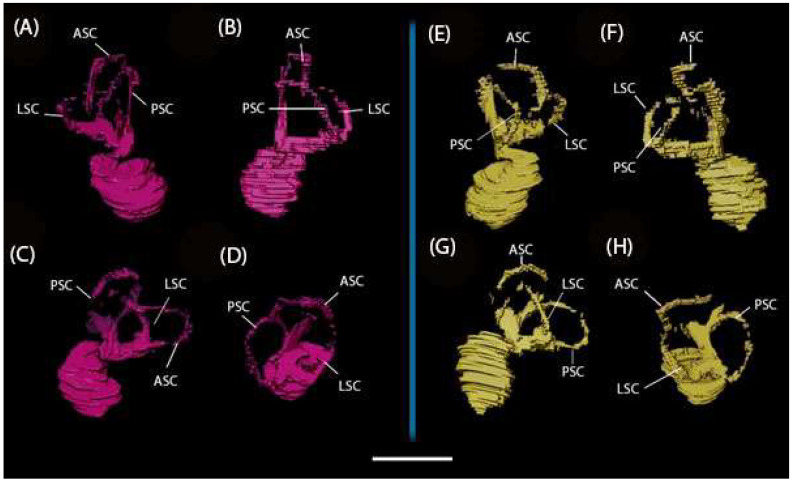
Reconstructed bony labyrinths of the holotype of *Mioeuoticus shipmani* (KNM-RU 2052). Left (pink) bony labyrinth in (**A**) posterior view; (**B**) anterior view; (**C**) inferior view; and (**D**) lateral view. Right (yellow) bony labyrinth in (**E**) posterior view; (**F**) anterior view; (**G**) inferior view; and (**H**) lateral view. Scale bar is 5 mm.

None of the canal reconstructions are complete, though it is unclear whether this is due to breakage or scan resolution (Figure 7 and Figure 8). The angles between all canals are far from orthogonal, with a large average deviation from orthogonality of 12.7° (Table 3).

The ASCs and PSCs have a degree of torsion, whereas the LSCs are fairly planar. The ASCs have the greatest degree of torsion, with the dorsal edge bent significantly forward. Of the SCCs, the ASC has the greatest height and width, and the PSC has the shortest (Table 4). The posterior limb of the LSC with the ampullar limb of the PSC fuse to form a secondary common crus orthogonal to the plane of the LSC length.

## 4. Discussion

### 4.1. Olfaction

A direct comparison between several modern and extinct lorisid olfactory systems, as well as that of the fossil prosimian *Rooneyia*, analysed in Lundeen & Kirk [38], is undertaken here to highlight the most prominent and remarkable characteristics of the reconstructed turbinals of *Mioeuoticus* (Table 5). Rooneyia is included as this taxon is the only proxy we have for what might be primitive more broadly in primates, but it is not a direct ancestor since it is generally recovered as a fossil haplorrhine.

The turbinals are described and compared from the relative posterior-to-anterior position in the olfactory system.

As aforementioned, *Mioeuoticus* has notably long, elongated maxilloturbinals. Compared to *Loris*, the MT/mt in *Mioeuoticus* is proportionally much longer than the other turbinals in the assemblage (though there seems to be anterior breakage of ET/et-I, and so it could appear reduced) (Lundeen [41]: Figure 9.2C,D). The maxilloturbinals of *Mioeuoticus* stand prominently anterior to the rest of the turbinals in the olfactory system. This contrasts the MT/mt of modern counterparts *Loris*, *Nycticebus, Arctocebus,* and *Perodicticus,* which sit almost directly under the other turbinals in the olfactory system (Lundeen [41]: Figure 9.2A–H). Thus, the modern counterparts have a more compact, overlaid arrangement than *Mioeuoticus*.

The MT/mt of *Mioeuoticus* and *Perodicticus* are broadly similar in relative surface area and shape; however, the turbinals in the *Mioeuoticus* specimen are too fractured to make a more detailed comparison. The maxilloturbinal total surface area (MTSA) of *Mioeuoticus* is a relatively small 151.36 mm^2^ in comparison to extant strepsirrhines and haplorhines studied in Lundeen & Kirk [38], e.g., the MTSA of *Rooneyia* is only 145.42 mm^2^, *Loris* is 221.20 mm^2^, *Nycticebus* is 221.74 mm^2^, and *Perodicticus* is considerably higher at 366.35 mm^2^ (the total maxilloturbinal surface area of all comparative primate specimens from Lundeen & Kirk [38] is available in Supplementary Table S4).

From the rostral-to-caudal limits of the turbinal assemblage of *Mioeuoticus*, the NT/nt has a similar relative surface area, position, scrolled structure, and slope gradient to that seen in *Arctocebus* and *Nycticebus* (Lundeen [41]: Figure 9.2A,B,G,H). The NT/nt is smaller than that of *Loris* and *Perodicticus*, with a shallower slope gradient on the sagittal plane. The NT/nt of these modern counterparts do, however, have a similar posterior position relative to the MT/mt in *Mioeuoticus* (Lundeen [41]: Figure 9.2C–F). The *NT/nt* of *Nycticebus* and *Perodicticus* contrast all of these specimens with a more anterior relative position to the MT/mt. There is a shallower slope gradient of the NT/nt on the sagittal plane in the olfactory system of *Mioeuoticus* than in that of *Nycticebus,* and significantly so than that of *Loris*, and the turbinal is not scrolled towards the posterior in these modern counterparts.

The FT/ft of *Mioeuoticus* are similar in relative surface area to *Nycticebus*, though with an arc structure rather than the scrolls seen in the modern counterpart (Lundeen [41]: Figure 9.2B). This arcuate structure is strikingly similar to that seen in *Rooneyia* (Lundeen & Kirk [38]: Figure 1F,G). The posterior of the FT/ft has an increasingly steep slope gradient towards the anterior in *Mioeuoticus* and *Rooneyia*, which mirrors that of the NT/nt. This characteristic is also evident, though much steeper, in *Nycticebus*. The FT/ft sits laterally to the NT/nt in *Mioeuoticus*, as opposed to a superomedial position in *Loris*.

The ET/et-I in the modern counterparts extend as far laterally as the MT/mt, whereas the ET/et-I of *Mioeuoticus* are in a significantly more posterior relative position, more posterior than the NT/nt too. ET/et-I are much larger in relative surface area in the modern specimens than in *Mioeuoticus* and *Rooneyia*. ET/et-I in *Mioeuoticus* are much smaller in relative surface area and length than in *Loris*, though these turbinals remain similar in relative size in each species. These ethmoturbinals also have weaker scrolled structures than those of *Loris* and *Arctocebus*, though it is difficult to determine if this is because of breakage in the latter. In *Mioeuoticus* and *Rooneyia,* the anterior margin of ET/et-I forms a mediolaterally compressed and laterally directed enclosed bulla that increases in volume anteriorly until it opens to a scroll structure (Lundeen & Kirk [38]: Figure 1E,F). ET/et-II and -III are strikingly similar in relative surface area, position, structure, and slope gradient on the sagittal plane in *Arctocebus*, *Nycticebus*, and *Mioeuoticus*, though much greater in surface area in *Loris* and *Perodicticus*.

The most striking feature in the olfactory system of *Mioeuoticus* is the location of the interturbinal. In lemuroids, ET/et-II originates from ET/et-I and/or the horizontal laminae and so the IT/it are located between the shared basal laminae of ET/et-I and -II. In contrast, in lorisoids, ET/et-II make contact with the lateral nasal wall directly and so the IT/it are located between ET/et-II and ET/et-III [38]. The IT/it of *Mioeuoticus* are unique in that, anteriorly, they are located between the shared basal laminae of ET/et-I and -II and ET/et-III, as seen in lemuroids and also in *Rooneyia* (Lundeen & Kirk [38]: Figure 1G). However, moving posteriorly on the coronal plane, ET/et-II contact the lateral nasal wall directly and so the interturbinal location changes. Notably, this is not between ET/et-II and ET/et-III but instead between ET/et-I and ET/et-II, so not the exact point of contact seen in modern lorisoids. This turbinal arrangement is not seen in any other known strepsirrhine, and the shared turbinal morphology with lemuroids likely denotes the primitive status of *Mioeuoticus* within Lorisidae, consistent with the idea that it might be a stem member of the family [14]. The IT/it of *Nycticebus* are similar in relative surface area to the rest of the assemblage, though it has a more elongated structure in *Nycticebus* than in *Mioeuoticus*. The IT/it of the olfactory system in *Loris* is similar in relative position to the IT/it of *Nycticebus.* However, it is more comparable in relative length to the IT/it in *Mioeuoticus* and much larger in relative surface area.

The most disparate characteristic in *Mioeuoticus* would be the absence of ET/et-IV, a feature which is present in *Nycticebus*, *Perodicticus*, *Arctocebus*, *Loris,* and *Rooneyia* (Lundeen [41]: Figure 9.2C,D; Lundeen & Kirk [38]: Figure 1H); though, as aforementioned, this ethmoturbinal is suspected to be present in *Mioeuoticus* and yet barely distinguishable due to the scan resolution. This is more likely when considering that the nasal fossae of strepsirrhines typically house 4–5 ethmoturbinals [38].

In several respects, there are close similarities in the organisation of the nasal turbinals between *Mioeuoticus* and *Rooneyia* [38]. The posterior of the NT/nt in *Mioeuoticus* and *Rooneyia* are connected to the superior wall of the nasal fossa via only the superior-most basal lamina (Lundeen & Kirk [38]: Figure 1F,G). The central portion of both NT/nt takes the form of a mediolaterally compressed scroll and the posterior-most portion of the NT/nt is more restricted in cross-sectional area and positioned in the superior nasal cavity, above the ET/et-I and superomedial to the FT/ft (Lundeen & Kirk [38]: Figure 1E). The NT/nt of *Rooneyia* contrast in that an anterior portion of the turbinals extends laterally to a more ventral position and then diverges posteriorly just anterolateral to the MT/mt (Lundeen & Kirk [38]: Figure 1C). Posteriorly, the ET/et of both *Mioeuoticus* and *Rooneyia* are branching structures that make contact with the superior and lateral fossa wall (Lundeen & Kirk [38]: Figure 1F–H), then the connection with the superior fossa wall is lost as the structure modifies into a singular bulla in the central and anterior portion of this turbinal (Lundeen & Kirk [38]: Figure 1B–E). The ET/et-II turbinals of *Mioeuoticus* and *Rooneyia* are inferomedial to ET/et-I, orientated anteromedially with a steep anterior gradient on the sagittal plane. It is difficult to discern whether the ET/et-III of *Mioeuoticus* are anteriorly bullar, like those of *Rooneyia* (Lundeen & Kirk [38]: Figure 1G), or scrolled, as this is not clear on the CT rendering. Posteriorly, the ET/et-III in both species curve more steeply to form a single medial scroll with branches that make contact with the lateral and anterior septal walls. The only considerable divergence is that *Mioeuoticus* has a much larger overall total surface area (OTSA) relative to body size (1109.92 mm^2^) than *Rooneyia* (408.31 mm^2^).

**Figure 9:**
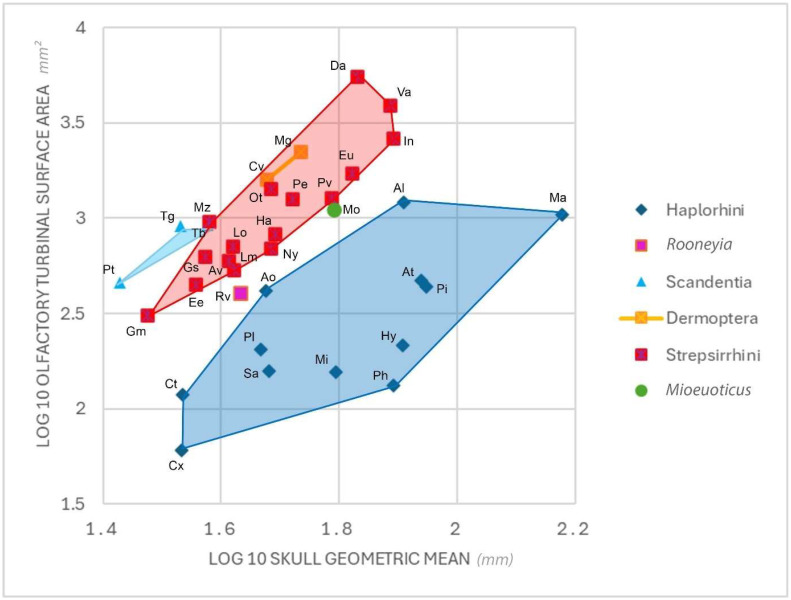
Biplot showing log-transformed olfactory turbinal surface area (measured in millimetres²) and log-transformed skull geometric mean (measured in mm). Skull geometric mean of cranial length and width is measured following Muchlinski [80]. The data used in this graph are modified from the supplementary information of Lundeen and Kirk [38]. These modified raw data are also available in Supplementary Tables S3 and S4. Taxon abbreviations: Al = *Alouatta*; Ao = *Aotus*; At = *Ateles*; Av = *Avahi*; Ct = *Carlito*; Cv = *Cynocephalus*; Cx = *Callithrix*; Da = *Daubentonia*; Ee = *Euoticus*; Eu = *Eulemur*; Ga = *Galeopterus;* Gm = *Galago moholi*; Gs = *Galago senegalensis*; Ha = *Hapalemur*; Hy = *Hylobates*; In = *Indri*; Lm = *Lepilemur*; Lo = *Loris*; Ma = *Mandrillus*; Mi = *Miopithecus*; Mo = *Mioeuoticus*; Mz = *Mirza*; Ny = *Nycticebus*; Ot = *Otolemur*; Pe = *Perodicticus*; Ph = *Presbytis*; Pi = *Piliocolobus*; Pl= *Plecturocebus*; Pv = *Propithecus*; Pt = *Ptilocercus*; Rv = *Roonyeia*; Sa = *Saimiri*; Tb = *Tupaia belangeri*; Tg = *Tupaia glis*; and Va = *Varecia*.

When comparing the total olfactory turbinal surface area relative to the cranial size of *Mioeuoticus* with that of extant and extinct primates in Lundeen & Kirk [38] study, it is evident that *Mioeuoticus* has an olfactory turbinal surface area that is broadly similar to the similar-sized strepsirrhines *Propithecus verreauxi*, *Euoticus elegantulus,* and *Perodicticus potto* (Figure 9). The OTSA of all comparative specimens from Lundeen and Kirk [38] and *Mioeuoticus* specimen KNM-RU 2052 are available in Supplementary Table S4. The surface area of *Mioeuoticus* is slightly smaller and plots just below the lower limit of strepsirrhine distribution. However, this is certainly influenced by underestimation caused by fragmentation and breakage.

*Mioeuoticus* has a notably extended olfactory recess length (7.53 mm). The presence of an olfactory recess is primitive for primates [77,78]. A large olfactory recess has been considered characteristic of mammals with a well-developed sense of smell [79] and haplorrhines are reported to lack an olfactory recess [37,60]. A larger olfactory recess results in greater odorant residence time, promoting slower airflow and improving the residence time of odorants within the olfactory region [77]. Further assessment of strepsirrhine airway morphometry and functional implications can be made by analysing the position of ethmoturbinals relative to the olfactory recess, e.g., Smith et al. [37]. However, as aforementioned, the resolution of the cranial scan in this study is too poor to fully determine the presence and extent of the fourth pair of ethmoturbinal in the *Mioeuoticus* specimen.

#### 4.1.1. Audition and Proprioception

Despite some features being undeterminable in the *Mioeuoticus* inner ear reconstruction, there are still key anatomical characteristics that give insights into the auditory capabilities and locomotor agility of the specimen, and also the phylogenetic status of the species.

**Figure 10:**
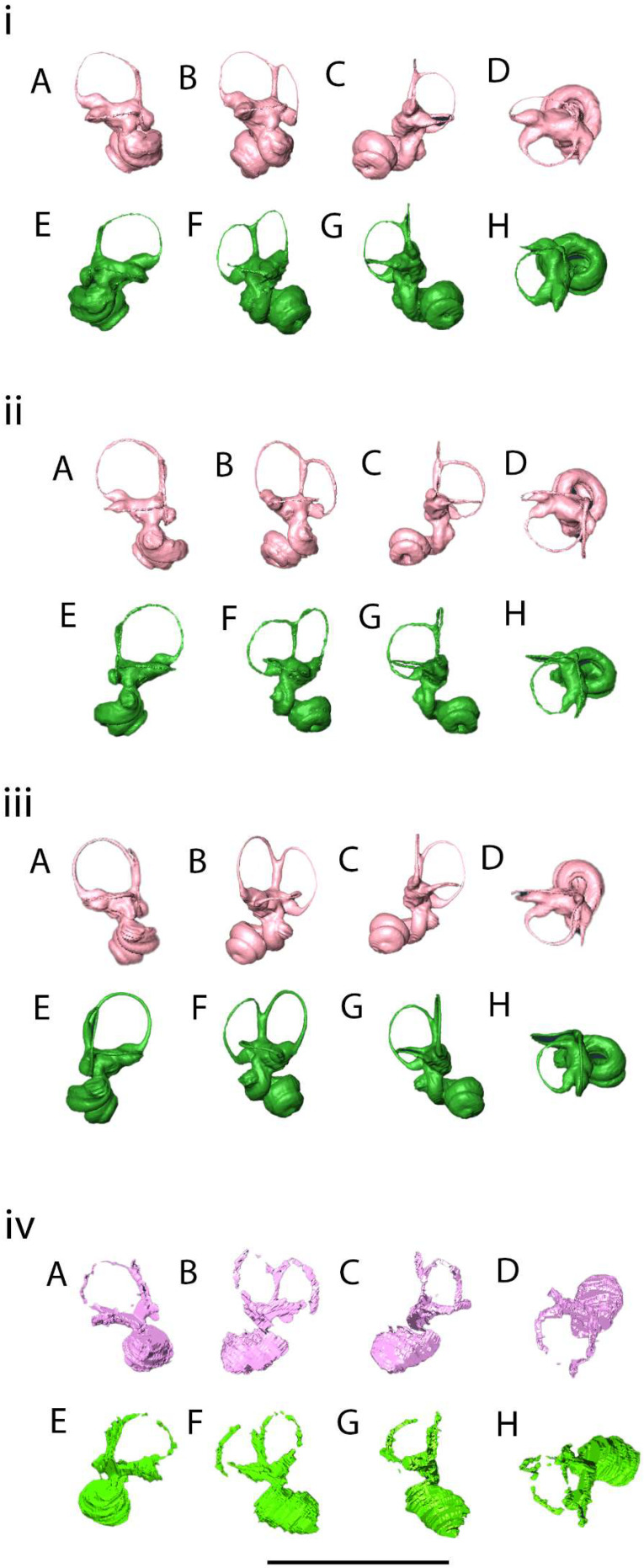
Digital visualisations of the (**i**) reconstructed left (pink) bony labyrinths of extant *Loris lydekkerianus* in (**A**) anterolateral view, (**B**) lateral view, (**C**) posterolateral view, and (**D**) dorsal view; reconstructed right (green) bony labyrinths of extant *Loris lydekkerianus* in (**E**) anterolateral view, (**F**) lateral view, (**G**) posterolateral view, and (**H**) dorsal view; (**ii**) reconstructed left (pink) bony labyrinths of extant *Nycticebus bengalensis* in (**A**) anterolateral view, (**B**) lateral view, (**C**) posterolateral view, and (**D**) dorsal view; (**ii**) reconstructed right (green) bony labyrinths of extant *Nycticebus bengalensis* in (**E**) anterolateral view, (**F**) lateral view, (**G**) posterolateral view, and (**H**) dorsal view; (**iii**) (i) reconstructed left (pink) bony labyrinths of extant *Perodicticus potto* in (**A**) anterolateral view, (**B**) lateral view, (**C**) posterolateral view, and (**D**) dorsal view; (ii) reconstructed right (green) bony labyrinths of extant *Perodicticus potto* in (**E**) anterolateral view, (**F**) lateral view, (**G**) posterolateral view, and (**H**) dorsal view; (**iv**) (i) reconstructed left (pink) bony labyrinths of *Mioeuoticus* holotype (KNM-RU 2052) in (**A**) anterolateral view, (**B**) lateral view, (**C**) posterolateral view, and (**D**) dorsal view; (ii) reconstructed right (green) bony labyrinths of *Mioeuoticus* in (**E**) anterolateral view, (**F**) lateral view, (**G**) posterolateral view, and (**H**) dorsal view. Scale bar is 10 mm.

The fusion of the anterior and posterior semicircular canals seen in the *Mioeuoticus* holotype is a common characteristic in numerous extant and extinct primates [81], and the orthogonality between this structure and the plane of the lateral semicircular canal is a condition that can also be observed in several primates, including Lorisidae [82]. The oval lateral canals and short common crura of the specimen are commonly exhibited by lorisoids, as opposed to the rounded lateral canals and long common cura which are indicative of Lemuroidea [83]. The *Mioeuoticus* cochlea is broad and bulky, similar in morphology to that seen in modern *Loris* (Figure 10i(A–H)) though significantly larger than *Nycticebus* and *Perodicticus* (Figure 10ii(A–H)). The common crus is short relative to the cochlea and semicircular canals, with a narrow utricle and vestibule, similar to that seen in *Nycticebus* (Figure 10iii(A–H)). The strong ovate shape of the semicircular canals is comparable between *Perodicticus* and *Mioeuoticus*, though the relative size between the canals is morphologically closer to that of *Nycticebus* (Figure 10iv(A–H)).

The 105° angle between the anterior and posterior semicircular canals in the *Mioeuoticus* specimen is remarkably consistent with the angles between the arcs at the greatest width of the anterior and posterior semicircular canals on the transverse plane (ASCm < PSCm) of platyrrhine and catarrhine anthropoids reported by LeBrun et al. [61]. According to this study, most primates exhibit an ASCm < PSCm much greater than 90°, and the platyrrhine and catarrhine anthropoids have an overlapping distribution spanning 101–110°. Following the current models correlating canal orthogonality and locomotor agility (e.g., [84,85]), we hypothesise that the significant deviation from orthogonality of the angles between all of the canals in the *Mioeuoticus* inner ear reconstruction may indicate slower rotational head speeds and a lower mean vestibular sensitivity to motion. These results are consistent with what would be expected for this taxon, but we take the opportunity to highlight this method has proven sometimes unreliable [86,87].

Walker et al. [7] previously measured the curvature radii in this specimen. The ASC radius is 2.6 mm, the PSC radius is 3.3 mm, and the LSC radius is 2.9 mm. The prediction for locomotor agility for fossil taxa based on a predictive equation for primates calculated from the values in Table 1 of Silcox et al. [88] predicts that animals with these value ranges are between 2 and 3 on a six-point agility scale (1 = extremely slow, 2 = slow [e.g., *Loris*], 3 = medium slow, 4 = medium [e.g., *Cebus*, *Cheirogaleus*], 5 = medium fast, 6 = fast [e.g., *Galago*, *Saimiri*]) formed by Spoor et al. [16] (e.g., [49,85,89]). Furthermore, the lateral canal has the shortest radius of the bony labyrinth in *Mioeuoticus*, and this characteristic is considered to be diagnostic of less agile behaviours [90,91].

The data in this auditory analysis of the *M. shipmani* holotype must be considered with caution, as several publications have determined that it is difficult to predict and differentiate vestibular sensitivities of slower-moving species from faster-moving ones when only a single specimen is available, as the morphologies of various individuals within a species and across several species overlap considerably (e.g., [66,90,91]). Recent studies indicate that the results acquired from measuring the orthogonality of the semicircular canals in any organism can lead to a fair amount of variability in results, particularly in a slow-moving taxon (e.g., Gonzales et al. [59]).

#### 4.1.2. Vision

**Figure 11:**
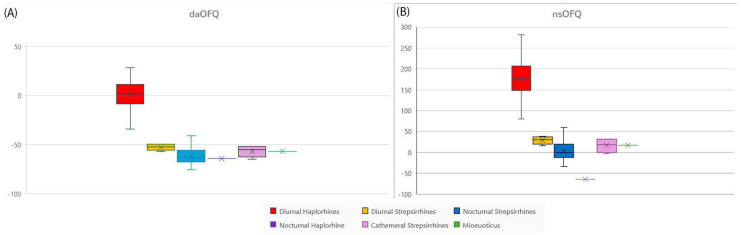
Quantile box plots comparing optic foramen quotients (OFQs) calculated for extant primates from Figure 9 of Kirk and Kay [21] compared to the OFQ of *Mioeuoticus shipmani* (KNM-RU 2052; holotype), using the diurnal anthropoid regression line ((**A**), daOFQ) and the nocturnal strepsirrhine regression line ((**B**), nsOFQ) from [21].

When making direct comparisons with the extant primate daOFQs and nsOFQs from Figure 11 in our study with the data in Kirk and Kay [21], the *Mioeuoticus* specimen has OFQs that fall well below the range of OFQs for extant diurnal haplorhines, borders the lowest range of OFQs for extant diurnal strepsirrhines (the lowest daOFQ being −56.9 for *Hapalemur griseus*), sits within range of extant nocturnal cathemeral strepsirrhines, and sits above nocturnal haplorhines (*Aotus*). The most similar OFQ values from the extant primates analysed in Kirk and Kay [21] are the diurnal strepsirrhine *Hapalemur griseus* (daOFQ = −56.9, nsOFQ = 16.40) and the nocturnal strepsirrhines *Galago matschiei* (daOFQ = −57.1, nsOFQ = 15.2) and *Perodicticus potto* (daOFQ = −57.2, nsOFQ = 15.5).

*Mioeuoticus* is known to be a strepsirrhine lorisid [1,5,14], and the OFQs calculated here have low values, which indicates a high central retinal summation, comparable to extant strepsirrhines with a nocturnal activity pattern. Thus, it is probable that *Mioeuoticus* had central retinas with a large proportion of high summation rods which increased sensitivity to light at the expense of visual acuity—an adaptation to low light levels.

The similarity in OFQs seen between this specimen and *Perodicticus potto* could suggest that these extant nocturnal strepsirrhines have retained a primitive optic morphology and, thus, nocturnal vision, as a generally plesiomorphic character state inherited from the ancestral *Mioeuoticus*. Accordingly, it may be surmised that this ancestral state character may closely resemble the visual acuity and optic morphology of these extant descendants.

## 5. Conclusions

Our research provides a unique new understanding of the reconstructed behaviour of *Mioeuoticus* and how the sensory sensitivities in this fossil lorisid compare to extant members of this family. Computed tomographic (CT) analyses of the sensory systems visible in the *Mioeuoticus* holotype specimen suggest it had a keen olfactory sense, less agile locomotion, and visual adaptations for either a nocturnal lifestyle, like that of modern lorisids, or a cathemeral lifestyle. Because the entire radiation of modern lorisids, as well as its sister group (Galagidae), are nocturnal, the most parsimonious explanation for the activity pattern of *M. shipmani* is that this animal belonged to an entirely nocturnal radiation of lorisoids. The unique turbinal arrangement is not seen in any other known strepsirrhine and shares a turbinal morphology with lemuroids, which likely denotes the primitive status of *Mioeuoticus* within Lorisidae. Our data are consistent with *Mioeuoticus* being a member of the family Lorisidae, as it shares morphological features in the olfactory system, orbital structure, and inner ear anatomy with modern representatives. Our results show that the behavioural pattern seen in the modern perodicticine lorisid *Perodicticus potto* most closely resembles that reconstructed in *Mioeuoticus shipmani*, and therefore *Perodicticus* could be used as an appropriate analogue for the early stages of sensory evolution in lorisids.

## Figures and Tables

**Table 1:** Anatomical measurements of turbinals in the left and right nasal fossae of the holotype of *Mioeuoticus shipmani* (KNM-RU 2052). All measurements are in mm. Lateral contact points are measured at the greatest degree of separation from superior and inferior contact points with the nasal fossae wall. Note these measurements are likely underestimates of turbinal size due to preservational issues.

	Left Nasoturbinal	Right Nasoturbinal	Left Frontoturbinal	Right Frontoturbinal	Left Interturbinal	Right Interturbinal	Left Ethmoturbinal I	Right Ethmoturbinal I	Left Ethmoturbinal II	Right Ethmoturbinal II	Left Ethmoturbinal III	Right Ethmoturbinal III	Left Nasolacrimal canal	Left Maxilloturbinal	Right Maxilloturbinal	Right Nasolacrimal Canal
Medial length	8.79	8.58	5.52	5.16	3.62	3.62	5.7	8.67	8.97	7.8	5.64	4.41	3.84	17.5	18.02	3.28
Lateral length	8.06	7.88	6.09	5.39	3.62	3.62	4.02	8.14	8.69	8.85	10.3	6.91	3.84	17.5	18.02	3.28
Anterior width	4.72	3.12	3.93	3.69	0.16	0.16	8.65	8.21	1.45	1.07	5.67	6.59	4.7	/	/	2.01
Posterior width	3.59	3.93	2.3	3.77	0.16	0.16	5.55	1.44	11.26	3.54	4.41	6.61	4.7	/	/	2.01
Scrolled portion	7.81	7.81	/	/	/	/	2.05	2.04	3.45	2.46	1.28	1.52	/	1.08	/	/
Scroll maximum diameter	3.32	2.57	/	/	/	/	2.19	4.59	2.4	2.57	3.7	4.94	/	3.5	/	/
Anterior height	3.87	2.73	5.73	4.84	1.28	1.28	4.61	2.98	9.1	3.12	2.35	6.52	/	/	/	/
Posterior height	12.13	12.57	1.29	1.74	0.05	0.05	0.85	0.95	2.74	1.48	2.76	2.57	/	/	/	/
Bulla portion	/	/	/	/	/	/	1.54	1.28	0.27	/	/	/	/	2.7	/	/
Bulla cross-section	/	/	/	/	/	/	3.22	7.02	2.4	/	/	/	/	3.5	/	/
Anterior lateral contact points	/	/	/	/	/	/	0.8	1.06	/	/	2.35	6.52	/	/	/	/
Posterior lateral contact points	/	/	/	/	/	/	/	/	/	/	1.63	1.73	/	/	/	/

**Table 2:** The total surface area of individual turbinal reconstructions and the overall turbinal assemblage of the holotype of *Mioeuoticus shipmani* (KNM-RU 2052). Measurements are taken in mm^2^.

Turbinal	Surface Area (mm^2^)
Maxilloturbinal (left)	151.36
Maxilloturbinal (right)	134.52
Nasoturbinal (left)	77.07
Nasoturbinal (right)	83.38
Ethmoturbinal I (left)	111.13
Ethmoturbinal I (right)	119.18
Frontoturbinal (left)	35.68
Frontoturbinal (right)	31.06
Ethmoturbinal II (left)	111.72
Ethmoturbinal II (right)	84.89
Interturbinal (left)	6.21
Interturbinal (right)	7.36
Ethmoturbinal III (left)	65.58
Ethmoturbinal III (right)	55.95
Fragments (left)	17.74
Fragments (right)	17.09
Total	1109.92

**Table 3:** The angle between the arcs at the greatest width of the anterior, posterior, and lateral semicircular canals on the transverse plane of the holotype of *Mioeuoticus shipmani* (KNM-RU 2052). Abbreviations: LSC—lateral semicircular canal, ASC—anterior semicircular canal, PSC—posterior semicircular canal. Measurements in degrees.

	LSC-ASC	LSC-PSC	ASC-PSC
Left inner ear	101°	101°	105°
Right inner ear	102°	102°	105°
Deviation from orthogonality	11.5°	11.5°	15°

**Table 4:** The height and width of the anterior, posterior, and lateral semicircular canals of the reconstructed bony labyrinths of the holotype of *Mioeuoticus shipmani* (KNM-RU 2052). Measurements in mm.

	Height	Width
Left		
Lateral semicircular canal	2.94	3.3
Posterior semicircular canal	2.34	3.05
Anterior semicircular canal	3.61	3.25
Right		
Lateral semicircular canal	2.93	2.92
Posterior semicircular canal	2.65	2.95
Anterior semicircular canal	3.38	3.19

**Table 5:** List of extant and extinct species used in the morphological and quantitative comparative analysis with respect to olfaction.

Species	Specimen ID	Publication Reference
*Nycticebus coucang*	MCZ-BOM-5118	Lundeen and Kirk [38]
*Loris tardigradus*	BAA-0006	Lundeen and Kirk [38]
*Perodicticus potto*	MCZ-25831	Lundeen and Kirk [38]
*Rooneyia viejaensis*	TMM 40688-7	Lundeen and Kirk [38]
*Arctocebus calabarensis*	YPM-Mam-014402	Lundeen [41]
*Mioeuoticus shipmani*	KNM-RU 2052	Current study

## Data Availability

The original contributions presented in this study are included in the article/Supplementary Material. Further inquiries can be directed to the corresponding author.

## Supplementary Materials

The following supporting information can be downloaded at https://www.mdpi.com/article/10.3390/ani15030345/s1, Supplementary Table S1: Combined measurements of the cranial length and the area of the optic foramen and the orbit of primate species analysed in Lundeen and Kirk [38] and this research study (as defined in Kirk and Kay [21]). These measurements are used to calculate the optic foramen index. P-I length: prosthion–inion length; OF index: optic foramen index. Supplementary Table S2: Calculations for the optic foramen quotient (OFQ) equation (as defined by Kirk and Kay [21]) as applied to *Mioeuoticus shipmani* specimen KNM-RU 2052. P-I length: prosthion–inion length; da: diurnal anthropoid; ns: nocturnal strepsirrhine. Numerical data from Supplementary Table S1. Supplementary Table S3: Measurements used to determine the log-transformed olfactory turbinal surface area of the primate species included in the biplot in Figure 9 (modified from Lundeen and Kirk [38]). Supplementary Table S4: Measurements showing log-transformed olfactory turbinal surface area, log-transformed maxilloturbinal surface area, and the log-transformed skull geometric mean from the biplot in Figure 10 (data derived from Lundeen and Kirk [38], with the addition of *Mioeuoticus shipmani* specimen KNM-RU 2052).
