# Supplementary material for: Sensory Reconstruction of the Fossil Lorisid Mioeuoticus: Systematic and Evolutionary Implications
Source: Animals (Basel). 2025 Jan 25;15(3):345. doi: 10.3390/ani15030345 (PMC11816023; doi:10.3390/ani15030345)
Supplement: Supplementary file 1 [file animals-15-00345-s001.zip › animals-3409725-supplementary.pdf]

Supplementary Information

Supplementary Table S1: Combined measurements of the cranial length, and the area of the optic foramen and the orbit of primate species analysed in Lundeen and Kirk [47] and this research study (as defined in Kirk and Kay [33]). These measurements are used to calculate the optic foramen index. P-I length: prosthion–inion length; OF index: optic foramen index.

Species	*Optic foramen area*	*Orbit area*	*P-I length*	*OF index*
Allenopithecus nigroviridis	7	298.7	98.5	2.33
Alouatta palliata	5.3	382.3	109.8	1.38
Alouatta seniculus	8	359.4	108	2.21
Aotus trivirgatus	3.1	297.4	60.5	1.05
Arctocebus aureus	1.2	134.8	49.5	0.89
Arctocebus calabarensis	1.6	156.6	53.8	1.01
Ateles fusciceps	9.7	469.4	115.1	2.08
Ateles geoffroyi	9.7	409.4	107.6	2.38
Atele paniscus	6.9	385.9	107.7	1.79
Avahi laniger	1.7	219.3	53.4	0.77
Cacajao melanocephalus	6.9	372.1	91.8	1.85
Callicebus moloch	3.5	163.9	59.7	2.14
Callicebus torquatus	3.8	192	65.8	1.99
Collimico goeldii	3.9	121.2	52.3	3.23
Callithrix argentata	2.8	77.3	45.2	3.68
Callithrix jacchus	3.2	72.4	43.7	4.39
Cebuella pygmaea	2.1	56.3	34.1	3.75
Cebus albifrons	7.5	328.7	87.5	2.31
Cebus capucinus	7.8	337	95.1	2.36
Cercopithecus cephus	9.3	344	95.9	2.73

Cercopithecus mitis	8.5	409.9	108.9	2.11
Cercopithecus petaurista	7.8	324.4	92.7	2.43
Cheirogaleus major	1.5	169.8	55.3	0.87
Cheirogaleus medius	1.1	117.9	43.3	0.94
Chiropates satanas	6.8	295.3	83.4	2.32
Chlorocebus aethiops	7.8	327.8	102.9	2.39
Colobus guereza	9.8	376.8	111.6	2.6
Colobus polykomos	8.5	391.2	111.1	2.2
Daubentonia madagascariensis	2.9	364.9	86.7	0.81
Erythrocebus patas	9.8	400.6	125.7	2.49
Eulemur fulvus	3.4	306.4	90.4	1.14
Eulemur macaco	3.1	295.8	89.5	1.06
Eulemur mongos	3	251.7	79	1.21
Eulemur rubriventer	3	317.7	86.2	0.96
Euoticus elegantulus	2.9	240	48.6	1.21
Galago alleni	2.3	211	49.6	1.08
Galago matschiei	3	201.1	45.2	1.49
Galago moholi	2.2	147.9	38.3	1.49
Galago senegalensis	2.6	183.9	43.9	1.45
Galagoides demidoff	1.5	109.2	37.3	1.36
Galagoides zanzibarus	2.1	125.8	40.8	1.7
Gorilla gorilla	15	1236	268.1	1.23
Hapalemur griseus	2.4	200.4	65.5	1.21
Hapalemur simus	4	340.4	81.9	0.88
Homo sapiens	14	721.1	186	1.88
Hylobates klosii	7.8	355.3	96.4	2.2
Hylobates lar	8.5	434.8	103.8	1.95
Hylobates syndactyles	9.7	447.6	123.7	2.22
Indri indri	4.5	427.6	103.3	1.06
Lagothrix lago	7	424.4	105.9	1.66
Lemur catta	3.2	258.9	83.5	1.24
Leontopithecus chrysomelas	3.1	112.5	57.3	2.78

Leontopithecus rosalia	2.9	110.3	55.1	2.65
Lepilemur leucopus	1	146	50.6	1.51
Lepilemur mustelinus	1.7	218.6	58.9	1.05
Loris tardigrades	2.8	216.8	48.7	0.84
Macaca fascicularis	12.7	392.7	109.7	2.09
Macaca mulatta	10.7	372.5	118.7	2.39
Macaca nemistrina	11.7	504.2	138.2	1.8
Microcebus murinus	1.3	77.6	32.8	1.86
Microcebus rufus	2.8	72.4	31.1	1.68
Miopithecus talapoin	3.7	227.1	72.8	2.93
Mirza coquereli	0.6	157	50.7	1.54
Nasalis larvatus	10.6	426.7	119.1	2.47
Nycticebus coucang	1.9	239.2	57.9	1.01
Nycticebus pygmaeus	0.4	212.9	52.4	1.2
Otolemur crassicaudatus	4.7	252.5	64.9	1.5
Otolemur garnetti	4.5	272.3	67.5	1.63
Pan troglodytes	9.6	899.6	187.1	1.58
Perodicticus potto	2.5	193.2	61.7	1.24
Phaner furcifer	2.2	166.9	52.4	1.63
Pithecia pithecia	3.4	215.3	75.9	2.34
Presbytis comata	2.8	396.1	92.8	2.22
Presbytis frontata	2.4	365.9	95.24	2.38
Presbytis melalophos	4.2	401.4	91.8	1.96
Procolobus badius	6.6	408.8	102.8	2.05
Propithecus diadema	4.4	392.5	89.4	1.11
Propithecus verreauxi	1.4	302.9	81.3	1.11
Pygathrix nemaeus	8	491.7	109.2	1.64
Pygathrix roxellana	9.4	454	113.2	2.1
Saguinus fuscicollis	3	80.2	45.2	3.77
Saguinus leucopus	3.2	78.4	47.8	4.08
Saguinus midas	3.6	77.3	49.4	4.68
Saimiri oerstedii	5.4	166.5	61.1	3.22

Saimiri sciureus	6.1	181.9	64.1	3.37
Semnopithecus entellus	9.9	413.6	117.8	2.41
Simias concolor	6.8	371.6	98.8	1.82
Tarsius bancanus	3.1	271.3	38.6	1.13
Tarsius spectrum	2.5	210.8	37.1	1.2
Tarsius syrichta	2.6	248.6	38.9	1.07
Trachypithecus cristatus	7	403.4	92.9	1.74
Varecia variegata	4	360.3	104.6	1.2
Adapis parisiensis	2.3	147.4	85.6	1.59
Adapis parisiensis	1.9	136.8	85	1.41
Adapis parisiensis	1.7	120.8	84	1.43
Adapis parisiensis	1.6	124.7	81.5	1.31
Leptadapis magnus II	2.8	373.3	119.2	0.75
Leptadapis magnus II	3.5	271.7	123.5	1.3
Leptadapis magnus II	3.6	376.6	106.1	0.95
Leptadapis magnus I	3.5	317.3	108.1	1.11
Leptadapis magnus I	3.5	314.2	95.5	1.13
**Microchoerus sp.**	1.7	141	46.3	1.21
Pronycticebus gaudryi	2.8	254.5	63	1.1
Rooneyia viejaensis	2.2	120.8	50	1.83
Rooneyia viejaensis	2.6	120.8	50	2.12
Plesiopithecus teras	2.5	229.7	52	1.07
Necrolemur antiquus	1.7	134.8	40.2	1.28
Necrolemur major	2	153.9	43.8	1.33
Simonsius grangeri	3.46	138.9	65.8	2.49
Arctocebus aureus	1.47	130.6	49.53	1.125574
Arctocebus calabarensis	1.67	155	56.79	1.077419
Euoticus elegantulus	3.4	251	47.62	1.354582
Loris lydekkerianus	0.85	467.5	52.27	0.181818
Nyctocebus bengalensis	3.79	254.4	65.61	1.48978
Nyctocebus coucang	2.18	316.15	65.52	0.689546

Nyctocebus pygmaeus	2.16	220.8	52.04	0.978261
Perodicticus potto	2.19	146.12	55.25	1.498768
Mioeuoticus	2.73	214.3	60.33	1.273915

Supplementary Table S2: Calculations for the optic foramen quotient (OFQ) equation (as defined by Kay and Kirk [33]) as applied to *Mioeuoticus shipmani* specimen KNM-RU 2052. P-I length: prosthion–inion length; da: diurnal anthropoid; ns: nocturnal strepsirrhine. Numerical data from Supplementary Table S1.

Optic Foramen Index (OFI) = (Optic Foramen Area/Orbit Area) * 100

(2.73/214.3) * 100 = 1.273915072

Diurnal Anthropoid Expected OFI: lnOFI = 3.39983-0.56624 * ln (P-I length)

60.33 = P-I length of *Mioeuoticus*

ln(60.33) = 4.099829492

lnOFI = 3.39983-0.56624*4.099829492 = 1.078342548

Expected OFI = $e^{1.078342548}$ = 2.939802929

(da)OFQ = (Observed OFI-Expected OFI)/Expected OFI] * 100

daOFQ = ((1.273915072-2.939802929)/2.939802929) * 100

daOFQ = -56.6666507

Nocturnal strepsirrhine Expected OFI: lnOFI = 2.46913-0.58138*ln(P-I Length)

60.33 = P-I length of *Mioeuoticus*

Ln (60.33) = 4.099829492

Expected OFI = $e^{0.085899116}$ = 1.08969639

nsOFQ = (Observed OFI-Expected OFI)/Expected OFI] * 100

nsOFQ = ((1.273915072-1.08969639)/ 1.08969639) * 100

nsOFQ = 16.90550541

Supplementary Table S3: Measurements used to determine the log-transformed olfactory turbinal surface area of the primate species included in the biplot in Figure 9 (modified from Lundeen and Kirk [47]).

Genus	*spec#*	*Mx Width*	*Mx Length*	*Area*	*√Area*	*Log(√Area)*
Alouatta_palliata	MCZ-5329	65.51	100.82	6604.7182	81.26941737	1.90992715
Aotus_griseimembra	MCZ-19802	38.8	57.97	2249.236	47.42611095	1.67601751
Ateles_geoffroyi	MCZ-5336	69.29	108.71	7532.5159	86.79006798	1.93847003
Avahi_laniger	MCZ-44879	35.8	49.01	1754.558	41.88744442	1.62208386
Callicebus_moloch	MCZ-26922	40.2	53.93	2167.986	46.56163657	1.66802824
Callithrix_argentata	MCZ-30582	28.58	40.93	1169.7794	34.20203795	1.53405198
Carlito syricta	DPC-045	32.03	36.79	1178.3837	34.32759386	1.53564336
Cynocephalus_volans	AMNH-M-187861	35.7	63.59	2270.163	47.64622755	1.67802852

Daubentonia_madagascariensis	AMNH-1006320	57.06	80.44	4589.9064	67.74884796	1.83090191
Eulemur_fulvus_collaris	MCZ-44896	49.7	88.63	4404.911	66.36950354	1.82196857
Euoticus_elegantulus	MCZ-14657	29.59	44.04	1303.1436	36.09908032	1.55749614
Galago_moholi	*MCZ-44132*	25.06	35.575	891.5095	29.85815634	1.47506299
Galago_senegalensis	DPC-007	30.27	46.27	1400.5929	37.42449599	1.57315596
Galeopterus_variegatus		42.5	69.83	2967.775	54.47728885	1.73621549
Hapalemur_griseus	MCZ-44913	40.95	59.1	2420.145	49.19496925	1.69192069
Hylobates_lar	MCZ-41463	66.82	97.67	6526.3094	80.78557668	1.90733383
Indri_indri	AMNH-M-100506	59.99	101.86	6110.5814	78.17020788	1.89304127
Lepilemur_mustelinus	AMNH-M-170568	33.94	49.53	1681.0482	41.0005878	1.61279008
Loris_tardigradus	BAA-0006	33.88	51.35	1739.738	41.71016663	1.62024192
Mandrillus_leucophaeus	MCZ-19986	111.95	202.62	22683.309	150.6097905	2.1778532
Miopithecus_talapoin	MCZ-23196	51.79	74.74	3870.7846	62.21562987	1.7938995
Mirza_coquereli	DPC-1139	30.36	47.58	1444.5288	38.00695726	1.5798631
Nycticebus_coucang	MCZ-5118	37.96	55.11	2091.9756	45.73811977	1.66027831
Otolemur_crassicaudatus	DPC-016	39.52	59.4	2347.488	48.45088234	1.68530169
Perodicticus_potto	MCZ-25831	44.51	62.52	2782.7652	52.75192129	1.72223828
Piliocolobus_badius	MCZ-24080	79.27	98.99	7846.9373	88.58294023	1.94735009
Presbytis_hosei	MCZ-37371	67.4	90.48	6098.352	78.09194581	1.89260624

Propithecus_verrauxi	MCZ-16375	50.34	74.99	3774.9966	61.44100097	1.78845828
Ptilocercus_lowii	USNM-481107	19.69	36.59	720.4571	26.84133193	1.42880406
Rooneyia		35.036	52.744	1847.938784	42.98765851	1.63334379
Saimiri_oerstedii	MCZ-10131	38.14	60.34	2301.3676	47.97257133	1.680993
Tarsius_syrichta	DPC-045	32.03	36.79	1178.3837	34.32759386	1.53564336
Tupaia_belangeri	USNM-320680	26.94	52.92	1425.6648	37.75797664	1.57700871
Tupaia_glis		25.57	45.18	1155.2526	33.98900705	1.53133848
Varecia_variegata_rubra	DPC-050	59.04	100.48	5932.3392	77.02168006	1.88661299

Supplementary Table S4: Measurements showing log-transformed olfactory turbinal surface area, log-transformed maxilloturbinal surface area, and the log-transformed skull geometric mean from the biplot in Figure 10 (data derived from Lundeen and Kirk [47], with the addition of *Mioeuoticus shipmani* specimen KNM-RU 2052).

Species	*Total surface area*	*Log10 total surface area*	*MT total surface area*	*Log10 MT total surface area*	*Log10 skull geometric mean*
Alouatta palliata	1217.09	3.085322694	283.93	2.453211282	1.909927146
Aotus grisimembra	418.29	2.621477482	144.1	2.158663981	1.676017513
Ateles geoffroyi	469.59	2.67171884	125.34	2.09808969	1.938470029
Avahi laniger	536.14	2.72927821	197.47	2.295501126	1.622083865
Callicebus discolor	205.24	2.312262006	177.97	2.2503468	1.668028237
Callithrix argentata	60.72	1.783331763	69.45	1.84167225	1.534051985
Carlito syricta	119.46	2.07722251	23.77	1.376029182	1.678028521

Cynocephalus volans	1601.71	3.204583887	138.77	2.142295588	1.830901915
Daubentonia madagascariensis	5516.88	3.741693538	516.27	2.712876889	1.821968569
Eulemur collaris	1713.84	3.233970275	1104.61	3.043208971	1.557496138
Euoticus elegantulus	447.12	2.650424097	163.92	2.214631945	1.475062988
Galago moholi	309.83	2.491123467	129.16	2.111128036	1.57315596
Galago senegalensis	625.91	2.79651189	282.18	2.450526229	1.736215486
Galeopterus variegatus	2233.34	3.348954844	221.46	2.345295296	1.691920693
Hapalemur griseus	828.34	2.918208634	424.74	2.628123163	1.90733383
Hylobates lar	214.81	2.332054495	610.63	2.785778137	1.893041267
Indri indri	2610.83	3.416778594	678.51	2.831556253	1.612790083
Lepilemur mustelinus	600.46	2.778484082	260.36	2.415574263	1.620241925
Loris tardigrades	713.62	2.853467013	221.2	2.344785123	2.177853204
Mandrillus leucophaeus	1041.53	3.017671784	1267.43	3.102923983	1.793899502
Miopithecus talapoin	156.77	2.195262958	134.83	2.129786535	1.579863102
Mirza coquereli	961.95	2.983152499	365.63	2.563041822	1.660278307
Nycticebus coucang	691.47	2.839773343	221.74	2.345844043	1.68530169
Otolemur crassicaudatus	1429.97	3.155326926	536.14	2.72927821	1.722238282
Perodicticus potto	1258.8	3.099956734	366.35	2.563896196	1.947350091
Presbytis hosei	133.29	2.124797568	126.75	2.102947968	1.892606244
Procolobus badius	436.93	2.640411865	215.98	2.334413537	1.947350091

Propithecus verreauxi	1266.9	3.102742336	878.78	2.943880164	1.428804063
Ptilocercus lowii	459.44	2.662228803	164.8	2.216957207	1.63334379
Rooneyia viejaensis	408.31	2.610990016	145.42	2.16262414	1.680992997
Saimiri oerstedii	158.9	2.201123897	80.43	1.905418069	1.535643363
Tupaia belangeri	918.41	2.963036604	333.23	2.522744093	1.577008713
Tupaia glis	907.61	2.957899272	185.17	2.267570627	1.531338477
Varecia rubra	3897.4	3.590774981	2134.4	3.329275812	1.886612988
